# Board of Health of New Orleans

**Published:** 1841-10

**Authors:** 


### BOARD OF HEALTH OF NEW ORLEANS.

The whole of the Mississippi Valley is interested in the sanatory regulations of New Orleans, as every neighborhood has its representatives in that city, from the end of autumn till the beginning of summer. It is remarkable, that till July last, the city of strangers should not have had an organized Board of Health. We are indebted to a friend for a series of newspapers, in which the arrangement for establishing such a Board, and some of its first proceedings are published. It appears from one of them, that to Dr. E. H. Barton, late a professor in the Medical College of Louisiana, the city is chiefly indebted for this important institution—of which, appropriately, he has been made the president. The ordinance establishing the Board was passed on the 9th of June. From the 12th of May to the 1st of December it is required to publish weekly statements of the number of deaths, with the age, sex, color, vocation, nativity, &c. of each individual; and from the 1st of December to the end of April, monthly reports of the same kind. When there is an epidemic disease, daily reports are to be made. We have before us some specimens of these reports, and think they promise to put us in possession of valuable information, on the statistics of disease in our great southern metropolis. In the opinion of the General Council of New Orleans, that city, with the exception of its summer and autumnal fevers, is as healthy, or even healthier than any other city in the Union; a conclusion which we are not disposed to controvert; but the new regulations, in a few years, will afford data for a correct decision. We congratulate Professor Barton on the successful result of his labors, and hope he will persevere. D.

